# “Being empathetic, being accommodating, not only to the person you’re talking to, but also to yourself“– a qualitative study on preparing and conducting interviews with palliative care patients

**DOI:** 10.1186/s12904-025-01769-4

**Published:** 2025-05-07

**Authors:** Chantal Giehl, Anastasia Suslow, Horst Christian Vollmar, Nino Chikhradze, Ina Carola Otte

**Affiliations:** 1 Department of Geriatric Medicine, Marien Hospital Herne, Ruhr University Bochum, Hölkeskampring 40, 44625 Herne, Germany; 2 Institute of General Practice and Family Medicine (AM RUB), Medical Faculty, Ruhr University Bochum, Bochum, Germany

**Keywords:** End-of-life care, Palliative care, Qualitative research, Qualitative interviews, Sensitive communication, Relationship of the communication, Vulnerability of study participants

## Abstract

This study explores the necessary considerations for preparing and conducting qualitative interviews with palliative care patients and their relatives. Palliative care patients are considered a vulnerable group, which raises ethical and methodological challenges for researchers. The study aims to address these challenges by emphasizing the importance of the relationship between researcher and participant, sensitive communication, and continuous self-reflection throughout the research process. Six semi-structured interviews were conducted with healthcare professionals in the field of palliative care. The results highlight four major themes that are constantly interacting and interrelated: relationship, communication, self-reflection and research framework. These factors are crucial in balancing the needs of participants with the demands of qualitative research. The findings suggest that a patient-centred, needs-oriented approach is fundamental to the collection of high-quality data, while simultaneously ensuring the well-being of both participants and researchers.

**Supplementary Information:**

The online version contains supplementary material available at 10.1186/s12904-025-01769-4.

## Introduction

When conducting research on, and especially with, vulnerable groups (e.g. palliative care patients), researchers need to adopt a sensitive approach [1]. In the field of palliative care, different research methods are used to collect and analyse scientific data [2]. Qualitative research being one of these and can identify and describe matters whose meaning is not known in advance of a research project. It can also raise questions about significance that cannot be quantified easily [3]. Furthermore, qualitative research is especially suited for palliative care settings as it allows for a deep exploration of emotional, existential, and lived experiences, which are often central to understanding the complexities of end-of-life care. These experiences may not be fully captured through quantitative methods. For this reason qualitative research can thus provide a holistic view of patients’ needs, values, and preferences [4]. Subjective views and motives for action can be captured at a deeper level [5]. It can also address fundamental issues, for example focusing on the life experiences of the participants. As a result, the questions asked in the interview can evoke strong emotional reactions [3, 6].

The field of palliative care is already considered vulnerable and can be additionally burdened by the presence of research. However, as Luna (2019) argues, vulnerability should not be seen as an inherent characteristic of specific groups but rather as a layered and context-dependent phenomenon. It arises from intersecting social, economic, and political factors, which may vary depending on the circumstances and individual experiences. Recognizing this complexity is essential to ensuring that ethical research practices do not reinforce static categorizations of vulnerability but instead address the specific challenges faced by individuals in palliative care settings [7]. Recent approaches in research ethics emphasize that vulnerability arises in relational contexts rather than being an inherent characteristic of a subpopulation. Researchers themselves may also experience vulnerability in qualitative health research, highlighting the need for reflexivity in the research process [8]. Palliative care patients and their relatives are therefore perceived as particularly vulnerable and in need of protection [2, 3]. Nevertheless, research in the field of palliative care is needed in order to gain further insights into the needs of patients and medical professionals and to further develop and adapt concepts on the provision of care. This creates an area of tension as to how the safety and protection of those involved can be ensured and how research can be carried out and new knowledge gained at the same time. In this context, ethical considerations are crucial. Kang and Hwang emphasize that researchers conducting qualitative research must adhere to key ethical principles such as ensuring informed consent, protecting confidentiality, privacy, honesty and integrity. These principles help ensure that vulnerable participants are respected and protected throughout the research process, and that the data collected is both ethical and valuable for scientific use [9]. Beauchamp and Childres additionally emphasize the obligation to avoid harm as one of the four central ethical principles [10]. Furthermore, maintaining the autonomy of the participants while at the same time balancing their need for protection poses a considerable ethical dilemma [10, 11]. However, Kendall et al. found most researchers to believe that research with palliative care patients is no more problematic than other research fields [12]. In addition, researchers are faced with the challenge of meeting the requirements of accurate data collection and balancing the needs of the participants. Challenges are for example the rapid fatigue and concentration of the participants due to their palliative condition. Being able to give detailed answers can also be a challenge for palliative care patients, although these are needed for scientific analysis [13].

To collect qualitative data in the field of palliative care, well-trained interviewers with in-depth methodological knowledge are necessary [3]. In qualitative research, particularly in palliative care, the relationship between the researcher and participant plays a crucial role in shaping the data. Building rapport can lead to more open and meaningful exchanges, yet the emotional vulnerability of participants requires the researcher to handle interactions with great sensitivity. This relationship may also influence the depth and quality of the data collected, as participants are more likely to share their personal and emotional experiences when trust is established [14]. However, qualitative researchers are often not trained to deal with “difficult” topics such as dying, death and grief, which is why new approaches are required for preparing and conducting such data. The palliative medicine section of the German Network for Health Services Research (DNVF) developed a memorandum discussing the specifics of research of and with palliative care patients. In the area of qualitative research, it states that researchers should undergo extensive interview training in order of preparation. In addition, comprehensive methodological knowledge is necessary for flexible handling of data collection [2]. Questions remain as how these aspects should look in concrete terms and, above all, what this means for researchers who enter the field of palliative care for the first time.

This publication therefore raises the question of which concrete aspects need to be considered when preparing and conducting qualitative interviews with palliative care patients and their relatives.

## Methods

In preparation for an exploratory research project with palliative care patients and their relatives, part of the research group took part in relevant training courses and decided to collect additional data on the topic of sensitive communication and interviewing to gain a deeper understanding. The data collection and examination of the topic was the first step of the research project to gain a deeper understanding and prepare the researchers for the following data collection in the main project with palliative care patients.

A qualitative design has been chosen for this part of the study. It consists of six qualitative, semi-structured interviews, focusing on sensitive communication and interviewing, preparation and reflexivity in conducting interviews with vulnerable groups taking the example of palliative care patients and their relatives.

### Sampling and data collection

The main focus of our sampling strategy was on people with close professional contact with the target group, as their day-to-day work brings them into regular contact with vulnerable people. They are experienced in delivering bad news and partly in conducting interviews, and they could provide realistic and practical insights into sensitive interviewing. Among other reasons, the interviews were conducted to approach the topic of sensitive conversations, which is why subjects who had no previous experience with qualitative interviews were also interviewed. Therefore, in order to identify suitable interview partners, only people who interact with palliative care patients and their relatives in their daily working lives were recruited. Firstly, the research team asked the professional association of palliative care physicians in Westphalia-Lippe for recommendations for interview partners. After receiving the recommendations, potential participants were contacted by mail and interview appointments were arranged. In addition, the sampling technique of snowballing was used by asking the participants for further potential subjects. This was important to ensure that the further participants belonged to a different professional group so that different perspectives could be included in the data collection.

Between April and May 2022 six semi-structured interviews (one in person, three via telephone and two via video call using Zoom), approximately 45–60 min in length, were conducted by CG and AS with each participant individually. The original interview topic guide is in German, as the interviews were conducted in German. For comprehensibility of the methodology, the English translation can be found in the Supplementary Materials.

### Data analysis

All interviews were transcribed verbatim and reviewed independently by CG and AS. The researchers carried out an independent analysis of all transcripts using MAXQDA 2022 software while following the steps of Kuckartz’s content analysis [15]. First, the data was coded separately by CG and AS, moving from concrete sections of the text to a more abstract level. The procedure was inductive and deductive at the same time. Meaning that the interview guide was used to initiate deductive coding, but new topics were also inductively included in the code tree. CG and AS then discussed the codes and code tree and recoded text passages again, if needed. In order to meet quality criteria for qualitative research, plausibility checks were carried out of each analysis of each interview. This allowed the researchers to reflect on their own background and potential bias (reflexivity) [16]. The encodings were then reviewed by a third independent researcher (IO) to ensure inter-rater reliability. All findings were discussed and critically tested in research group meetings. In this way, any discrepancies were resolved. As the study was conducted in German, for this paper CG translated the quotes and back-translated them to eliminate any confusion of meaning [17].

## Results

A total of six interviews were conducted with people who have daily interaction with palliative care patients and their relatives. The professions of the participants are physicians, coordinators, grief counsellors and social education specialists with a focus on palliative care. The age of the participants is between 27 and 64 years and two of them are male. Due to the small sample size, we are not able to disclose further characteristics, as their anonymity would be affected.

**Fig. 1:**
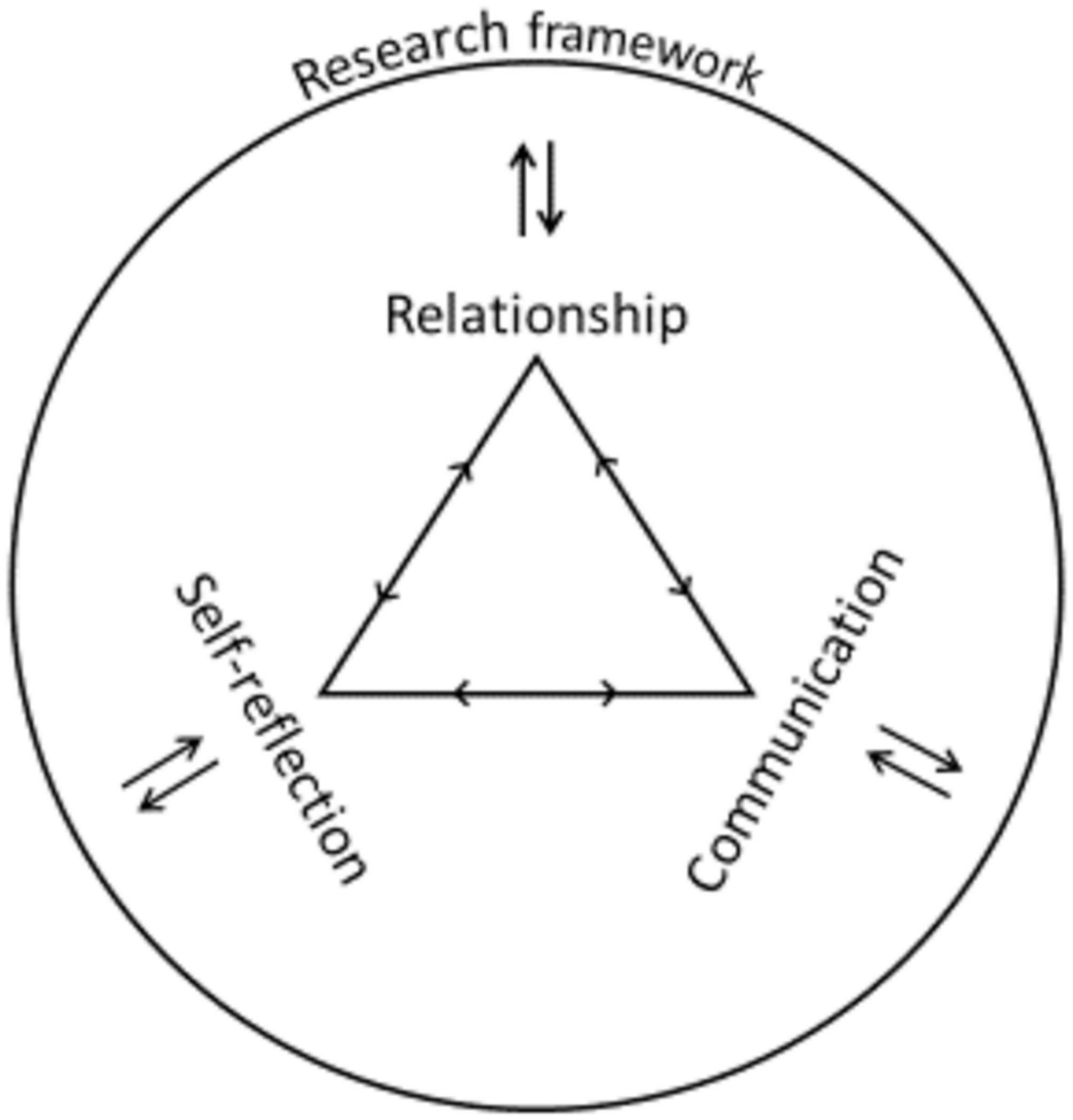
Main topics emerged from the analysis

The presented results consist of four major topics that emerged during the analysis of the interviews to ensure that the needs of palliative care patients and their relatives are met in qualitative research interviews and that a clean and high-quality data collection can take place at the same time. Those topics should be considered when preparing and conducting data collection. The topics relationship, communication and self-reflection were developed from the interview data and are based on the previously established interview guide. The fourth theme, the research framework, results from the additional reflection on the results in connection with knowledge and scientific literature on the research process. In general, the interviewed participants have no reservations about offering palliative care patients and their relatives the opportunity to participate in scientific research– as long as the following points are considered in the research project. The three main topics of relationship, communication and self-reflection are of equal and interrelated importance in this context. In addition, the research framework (fourth topic) embeds these aspects and clarifies the overall range of external factors and preparatory measures that structure and support the research process. For a better overview of the topics, see the following figure (Fig. 1).

### Relationship

In qualitative research, the relationship between the researcher and the participant is of particular importance, especially when the topic is sensitive, such as in palliative care. A relationship of trust allows for more in-depth and honest conversations, which increases the quality and depth of the data. In addition, a good relationship helps to maintain ethical standards and treat participants with the necessary respect and empathy.

Trust is the foundation of any successful interaction, especially in sensitive contexts such as palliative care. To establish this trust, a researcher’s willingness to demonstrate openness, honesty, and an appreciation for the gravity of a patient’s situation is dependent. A fundamental element of this is the active involvement of the patient in the research process. This implies that the patient’s viewpoint is consistently prioritised, and their requirements and preferences are upheld. Such involvement serves not only to reinforce trust but also to facilitate the establishment of an authentic and collaborative relationship in which the patient feels secure and understood: „So I think it’s always about space, time and, in quotation marks, an understanding counterpart. In other words, open ears and an open heart” (grief counsellor).

In addition to building trust, it is important to recognise the patient as a whole and accept their needs. Researchers need to tune into the patient’s perspective and set aside their own point of view in order to truly understand them. When working with palliative patients, it is crucial to recognise and respect their individual needs and wishes. Needs-orientation means engaging with the patient’s perspective and understanding their ideas and wishes without imposing your own standards or ideas. Acceptance is the key here, as it requires accepting the patient in their entire situation and valuing their decisions and feelings: „I understand what his concerns and wishes are when I don’t start from my quality of life and not from my idea of his quality of life, but when I actually get to the level of communication where it’s really about him and not about me“ (physician).

Being able to respond appropriately to the patient’s needs requires empathy and sensitivity. Empathy involves putting oneself in the patient’s emotional position and understanding their feelings and concerns. Sensitivity, on the other hand, refers to the ability to deal gently with the patient’s emotional and physical needs without losing the necessary professional distance. This closeness enables the patient to feel understood and cared for, which is particularly important in sensitive situations. On the other hand, the researcher must maintain a certain distance in order to protect themselves emotionally and be able to act professionally. This distance is necessary to remain objective and limit one’s own emotional burden: „That you are not only empathetic with the other person, but also empathetic with yourself. In these sensitive conversations in particular, there are often points that are somehow close to you personally. […] So being empathetic, being accommodating. But not only to the person you’re talking to, but also to yourself“ (social education specialist).

One of the main ways of realising these aspects is through honest and respectful communication at eye level. As it has a significant influence on the relationship, its individual aspects should be looked at more closely.

### Communication

Communicating effectively is not just an exchange of information, but also a way of building trust, understanding needs and establishing an empathetic connection. Especially in sensitive and emotionally challenging situations, the way in which communication takes place can significantly influence the progress and outcome of the conversation.

Honesty, authenticity and equal communication are essential components of a successful and trusting interaction between researchers and participant. It is about communicating openly and honestly. This means that the patient is perceived as an equal partner in the dialogue and that their opinions, wishes and needs are taken seriously. Such communication promotes trust and ensures that the patient feels heard and understood. A respectful exchange at eye level creates an environment in which the patient can express their thoughts and feelings openly and honestly without feeling ignored or patronised: „I think it’s important to be open and honest and tell the patient when they say it’s so bad at the moment that it is bad, and not necessarily sugarcoat it. That there is simply honest communication” (coordinator).

As communication does not occur exclusively on a verbal level, non-verbal communication should also be considered. It includes all forms of communication that take place without words, such as facial expressions, gestures, posture and eye contact. These subtle signals can often reveal more about a person’s inner attitude and emotions than spoken words: “I pay attention to the signals that the person sends me, to their emotional situation, their mood, their wishes, their concerns. I ask them questions. I take breaks in the conversation. I try not to dominate the conversation, but to give them the space they need” (physician). It goes without saying that researchers communicate things about themselves through non-verbal communication as well. Since communication always goes in two directions, the researcher should also pay attention to his own signals.

When non-verbal signals are perceived, it is up to the researcher to react flexibly and spontaneously. They enable an appropriate response to unexpected situations and emotional reactions. Flexible communication means being able to deviate from the planned course of the conversation in order to respond to the current needs and emotions of the other person. This applies both in terms of content and form. Once again, this shows a balance of needs-orientation and good data collection: „I have an internal structure of what I want to know and yes, then I think ahead, but of course I answer or respond to what the patients say. And that’s just so important or so interesting […] and you have to find a compromise somehow so that both of us come out of it well” (physician). At the same time, effective non-verbal communication also requires a high level of sensitivity and awareness of one’s own emotional reactions and boundaries. This is where self-reflection becomes a key element in the process.

### Self-reflection

Only through good self-reflection before, during and after such conversations can high-quality data collection succeed while considering and respecting the needs of all involved. As preparation and follow-up, researchers should reflect on their own experiences with the topic. This is a continuous process and should not only be realised before a study is conducted.

A fundamental palliative attitude can be developed through the process of coming to terms with one’s own finiteness and experiences within this context. It should be noted that this is not a fixed state of being; rather, it is constantly evolving: “But I believe that most, or hopefully all, of those who work in these areas are, on the one hand, very professionally competent and qualified and have hopefully all dealt with their own experiences and experiences that have to do with farewell, grief and death. And if they have done that, […] they have developed such an attitude for themselves, and it can’t be detached from themselves” (grief counsellor). This attitude is mentioned in several passages, as it has a fundamental influence on the entire research process.

Furthermore, the process of self-reflection occurs concurrently while collecting data. Through the awareness of one’s own attitude and perceptions, one is faced with the challenge of endurance. Since reality does not always go hand in hand with what one wishes for or perceives as good, in the research process it also applies to support and endure the participant’s situation and emotional world: “And many of us have an idea of what is beautiful and what is not and what is reasonable and what is not. Everyone has their own idea, and it is often the case that we differ greatly from this and that you have to endure it personally - that was actually a learning process for me, and I don’t think it happens overnight. […] And I also accept that the situation cannot be changed at the moment because that is what the person wants” (coordinator). In order to remain able to act appropriately in such situations, researchers should consider in advance how they would deal with other views and react to them.

In addition to their own palliative attitude, researchers are also required to pay attention to their own emotions and practise mental hygiene. This can be realised by exchanging experiences and views with colleagues in the research team and by reflecting on one’s own needs, such as taking a walk after the interview.

### Research framework

All the factors mentioned are influenced by external and preparatory measures that are intended to structure and support the research process. The research framework comprises structural and organisational aspects of the research, including the temporal and geographical context as well as preparation and follow-up.

Since the centre of the temporal context consists of flexibility and orientation towards individual needs, the health condition and daily routine of the palliative care patients should be considered when planning the interview times. This also influences the planned duration of the interviews, as it is better to plan additional backup time. On the one hand, greater time capacity means that the interview can take place in a calm atmosphere and the researcher is not rushed. On the other hand, with this flexibility the researcher can integrate breaks as well, when needed: “You notice that the pauses are getting longer or, or, or. That you then just say briefly that we’re going to take a break or simply ask the other person: “How is it for you right now?” Should we take a break? Should we perhaps pick it up again at another time?” (social education specialist). Nevertheless, a broad time frame should be set in advance to obtain a realistic expectation among the parties.

Regarding the geographical context, it is most important that the participant feels safe. The conversation should take place at a location without disruption and for this reason, and to create and maintain a basis of trust, a door can be closed, for example. In most cases, conversations with palliative care patients take place in their own homes. However, this information is particularly useful if the conversation takes place in a different location, such as a hospice: “So I think that the environment should be like this and I always try to ensure that the conversations I have are in a closed room or a closed area where you can actually achieve confidentiality so that outside influences don’t interfere with the conversation” (physician). One participant even named the seating position as a factor, that should be considered. Here too, there is no right or wrong and each person should figure out for themselves whether they prefer e.g. to sit with their back to the door, sideways next to the participant, face-to-face in front of them or in any other way. This example shows that it is often small aspects that can have a big impact on the well-being of those involved in the conversation and consequently on the result of the data collection.

The personal preparation of the researchers for the interviews is also a component of the research framework. Directly before an interview, researchers should check in with themselves and pay attention to their own needs. By fulfilling them in advance, the researcher can concentrate on their interview partner. This kind of ritual can help to approach the situation with calm and a clear mind: “Of course, I have to pay attention to how I’m doing personally. I have to develop rituals to take my time. I also mean making time mentally for this conversation, for this patient. In other words, I have to try to put everything else aside and concentrate only on the patient in this conversation, in this communication.” (physician). Other examples of rituals mentioned in the interviews are taking a deep breath, going for a short walk or wearing certain clothes.

Rituals also play an important role in the follow-up. First of all, the conversation should be documented. A standardised procedure is usually used for this, especially in the context of research. However, the individual’s own feelings should also be reflected and documented in this context. Also, after the interview, the researchers should check in with themselves and consider what is needed. Since the content and context of such interviews can be exhausting for the researcher, self-reflection and orientation towards one’s own needs are also relevant here. It is important to find out whether it is different things, such as a phone call with colleagues or a short walk, or whether the researchers establish a ritual that they carry out after the interviews. For example, one participant mentions: “And I actually have an outlet, I sit in the car afterwards and always like to listen to loud music (laughs). That’s my outlet to relax” (coordinator).

Not only after the interview, but also in other situations, it is important to exchange experiences with colleagues or to ask for collegial supervision. Especially when it comes to sensitive topics that are discussed in interviews with palliative care patients or the situation itself. Exchange with colleagues and the research leader must be ensured throughout the research process. This is the only way to ensure structural security for the researchers and, as a result, for the palliative care patients and their relatives.

## Discussion

The qualitative interviews explored aspects need to be considered when preparing and conduction qualitative interviews with palliative care patients and their relatives. The presented findings can be divided into three main topics, which are equally related to each other and influence and determine each other. Due to the strong interweaving of the main topics, a clear separation is only possible to a limited extent. For example, content such as needs-orientation can be found in all three topics.

The relationship between the researcher and the participant is one of the most relevant differences between qualitative and quantitative research [18]. The qualitative research process depends on it, because the relationship and the associated aspects, such as trust, have a major influence on the extent to which the participant opens up and allows the researcher to enter their world. Here, researchers are faced with the challenge of finding and maintaining a balance between professional closeness and distance. In this context, Halpern explored the role of empathy in the relationship between patients and physicians [19]. She argues that empathy is a crucial component of effective patient care and highlights that it requires physicians to balance emotional attunement with professional distance, ensuring they understand patients’ emotions without becoming overwhelmed. The paper recommends that physicians cultivate an empathic stance that is both authentic and sustainable, recognizing their emotional limits while still providing compassionate care [19]. These conclusions can also be applied to the situation of researchers. In the field of palliative care, they too have to balance their emotional reactions in order to maintain a good mix of distance and closeness. In contrast, a study by Kendall et al. states that the emotional challenges associated with qualitative research in palliative care are no greater than those in other areas where qualitative research is conducted [12].

Maintaining this balance goes hand in hand with honest communication at eye level. Communication should be authentic and adapt flexibly to unpredictable situations. Since the emotional and physical state of patients is always of crucial relevance, researchers should approach them with a sense of sensitivity, mindfulness and respect at all times [12]. In addition to the content, researchers should also pay attention to non-verbal signals and react flexibly. Halpern asserts that physicians demonstrate awareness of non-verbal communication by promptly modifying their gestural, pausing, and interpersonal distance behaviours in conjunction with the patient [19]. It is particularly challenging for researchers when situations affect them emotionally. This is possible since researchers are also human beings with own experiences. They should therefore reflect on them before entering the field of research and approach the situation in a stable state. To be able to react flexibly, researchers should be well trained and avoid additional stress for patients [3]. However, it must also be noted that it is questionable how well one can prepare for such situations and to what extent the experience will be significant.

As outlined in the previous paragraphs, self-reflection on the part of the researcher is another fundamental element of qualitative research in palliative care. Researchers should reflect on their own experiences before and after data collection and develop a fundamental palliative attitude. This process is ongoing and by no means static. This means that even during data collection, the researcher should actively reflect him- or herself [20]. Olmos-Vega and colleagues also supplement this aspect with the content of expectations, assumptions and conscious and unconscious reactions [21]. Reflection thus becomes larger and involves more than reviewing previous experiences. In this context, emotionally challenging situations can arise and can only be endured and carried together with the patient through a stable fundamental attitude. Exchange with colleagues can provide support during preparation and follow-up and contribute to the researchers own mental hygiene. Sterie et al. emphasize that researcher vulnerability is an important aspect to consider, particularly in emotionally demanding fields such as palliative care. To mitigate emotional burden, they recommend conducting data collection in pairs, organizing debriefing and supervision sessions, and engaging in ethical reflections before and after fieldwork [8]. Moreover the encouragement and support of team colleagues is an essential component in the development of coping strategies and competences of people working in the field of palliative care [22]. Another important aspect is that, in addition to the needs of the patients, attention should also be paid to the researchers needs during the entire research process. Practical measures, such as piloting data collection, adapting research protocols as needed, and fostering participatory research approaches, can help researchers navigate ethical and emotional challenges [8]. An easy-to-use tool for reflection before, during and after data collection is the practical guide to reflexivity in qualitative research by Olmos-Vega et al. [21].

The topics relationship, communication and self-reflection are embedded in the research framework and are in a constant interrelationship with each other and with the research framework. The results show that patients should feel comfortable in the facilities during the interview and their confidentiality and privacy are respected, especially as it is one aspects of ethical conducts in qualitative research [9]. Time flexibility also plays a role in this context and should be considered by researchers when planning and conducting data collection. For practical reasons, however, you also have to make sure that a certain time frame is not exceeded. Data collection is only one part of a researcher’s tasks and therefore cannot take up an unlimited amount of time [23]. In addition to time capacities, financial budgets of studies must also be managed. The interviews are usually transcribed after recording so that they can be analysed. As this process is often carried out by external transcription companies, a further cost factor arises here if the interview duration is significantly longer than originally planned. It is important for the research team to calculate well in advance and strike a balance between the requirements of a feasible study and the needs of the patients at the same time [24].

It is obvious that needs-orientation, trust, acceptance and empathy run through all topics, making it difficult to clearly separate the mentioned aspects. It becomes clear that all aspects are closely interwoven and constantly influence each other. Researchers’ fears that patients are already vulnerable and will be further burdened by their participation in research [3] can be countered by taking these aspects into account. Overall, the aspects mentioned show a great field of tension between the challenges of (qualitative) research and the challenges found in palliative care. A balance needs to be found and maintain in order to carry out high-quality data collection and at the same time meet the needs of palliative patients and their relatives.

Like most studies, this research is subject to some limitations. The sample size is not very large, which limits the generalisation of the results. However, a saturation of content has been achieved. In addition, all interviewed participants are healthcare professionals who are in daily contact with palliative care patients. They are not researchers themselves and can only provide information from their perspective. There may also be a selection bias, as the participants have a positive attitude towards research. This can be assumed as they took part in this research study and that may not reflect reality.

## Conclusion

The fundamental requirement for preparing and conducting research activities in the context of palliative care is an active confrontation with the (own) finiteness of life in order to develop a fundamental palliative attitude and to achieve constant self-reflection on the part of the researchers. This can be achieved through targeted education and training in palliative care and communication skills. Further aspects that need to be considered are the relationship between researcher and patient, the communication and the surrounding research framework. It can be concluded that researchers are primarily faced with the challenge of creating a good balance between the requirements of qualitative research and the requirements of meeting the patients’ needs. The key aspect in this context is needs-orientation– the needs of the patients but also those of the researchers.

## Electronic supplementary material

Below is the link to the electronic supplementary material.

Supplementary Material 1

## Data Availability

The data generated and analysed during the study are not publicly available to maintain participants’ privacy but are available from the corresponding author on reasonable request.
