# Supplementary material for: “Being empathetic, being accommodating, not only to the person you’re talking to, but also to yourself“– a qualitative study on preparing and conducting interviews with palliative care patients
Source: BMC Palliat Care. 2025 May 7;24:132. doi: 10.1186/s12904-025-01769-4 (PMC12057236; doi:10.1186/s12904-025-01769-4)
Supplement: Supplementary file 1 — Supplementary Material 1 [file 12904_2025_1769_MOESM1_ESM.docx]

**Interview topic guide for sensitive conversations**

**Topic I: Personal experiences**

**General introduction**

- What is important to you personally when you talk to people at the end of life?

**Sensitive conversation**

- Please tell me what you understand by the term "sensitive conversation".
  - Why is communication important at the end of life?
  - In which situations should you pay attention to sensitive conversation?
    - How does this work in your day-to-day work?
  - What do you pay attention to in such a conversation in order to meet the needs of people in the last phase of life?

**Experience of sensitive conversations**

- With what aim do you have conversations with people in the last phase of life?
  - What do you pay particular attention to?
- In your opinion, what does conversation at the end of life depend on?
- What qualities should an interviewer have?
- How do you prepare yourself for a conversation? Or with what attitude do you go into a conversation?
  - Do you take notes?
  - Do you have some kind of ritual or similar that you do beforehand?
- How do you prepare for the conversation?
  - Do you talk to colleagues about specific conversations? (Keyword: supervision)
- Do you have an example of a particularly successful conversation?
  - Why do you think it was like that?
- Do you have an example of a conversation that was not/rather less successful?
  - Why do you think it was less successful?
  - Were you able to save the conversation? If yes: How? (Strategies used)

**Topic II: The conversation**

- To what extent do you bring your own views on sensitive topics into the conversation?
  (e.g. spirituality, religion, life after death, funeral)
- Which relevance does the environment in which the conversation takes place have?
- How much time do you invest in such a conversation?
  - Do you set a fixed time frame in advance?
  - What happens if the time frame is (significantly) exceeded?
- Are there any topics that are raised particularly frequently? By whom?
  - Can you imagine the reasons why these topics come up frequently?
  - Which topics tend not to be addressed? Why?
- Which topics do you actively avoid in conversation?
  - Why do you avoid these topics?

**Topic III: Outlook**

- Did you have any training or education to prepare you for such conversations?
  - If yes: How did you feel about this training? What was particularly helpful?
  - If no: Would you have liked a training course and what content would be important to you?
- What significance do such conversations have for you personally?
  - Examples: Other attitudes to life? Living in the here and now? Personal attitudes towards the dying process, death, etc.

**Conclusion and acknowledgement**

- Do you have anything else that we haven't talked about yet, but that is still on your mind?
- Thank you for your time and your willingness to take part in our study

The original interview topic guide is in German, as the interviews were conducted in German. For comprehensibility of the methodology, therefore it is translated into English.
